# Supplementary material for: The separation pin distinguishes the pro– and anti–recombinogenic functions of Saccharomyces cerevisiae Srs2
Source: Nat Commun. 2023 Dec 8;14:8144. doi: 10.1038/s41467-023-43918-4 (PMC10709652; doi:10.1038/s41467-023-43918-4)
Supplement: Supplementary file 1 — Supplementary Information [file 41467_2023_43918_MOESM1_ESM.pdf]

The separation pin distinguishes the pro– and anti–recombinogenic functions of *Saccharomyces cerevisiae* Srs2

Aviv Meir[1†], Vivek B. Raina[1†], Carly E. Rivera[1], Léa Marie[2,4], Lorraine S. Symington[2,3] and Eric C. Greene[1]

[1]Department of Biochemistry & Molecular Biophysics, Columbia University, New York, NY, 10032, USA
[2]Department of Microbiology & Immunology, Columbia University, New York, NY, 10032, USA
[3]Department of Genetics & Development, Columbia University, New York, NY, 10032, USA
[4] Present Address: Institute of Pharmacology and Structural Biology (IPBS), French National Centre for Scientific Research (CNRS), Université Toulouse III, Toulouse, France
[†]Equal contribution.

To whom correspondence should be addressed: ecg2108@cumc.columbia.edu

Includes:

Supplementary Figures 1 through 5

Supplementary Tables 1 through 4

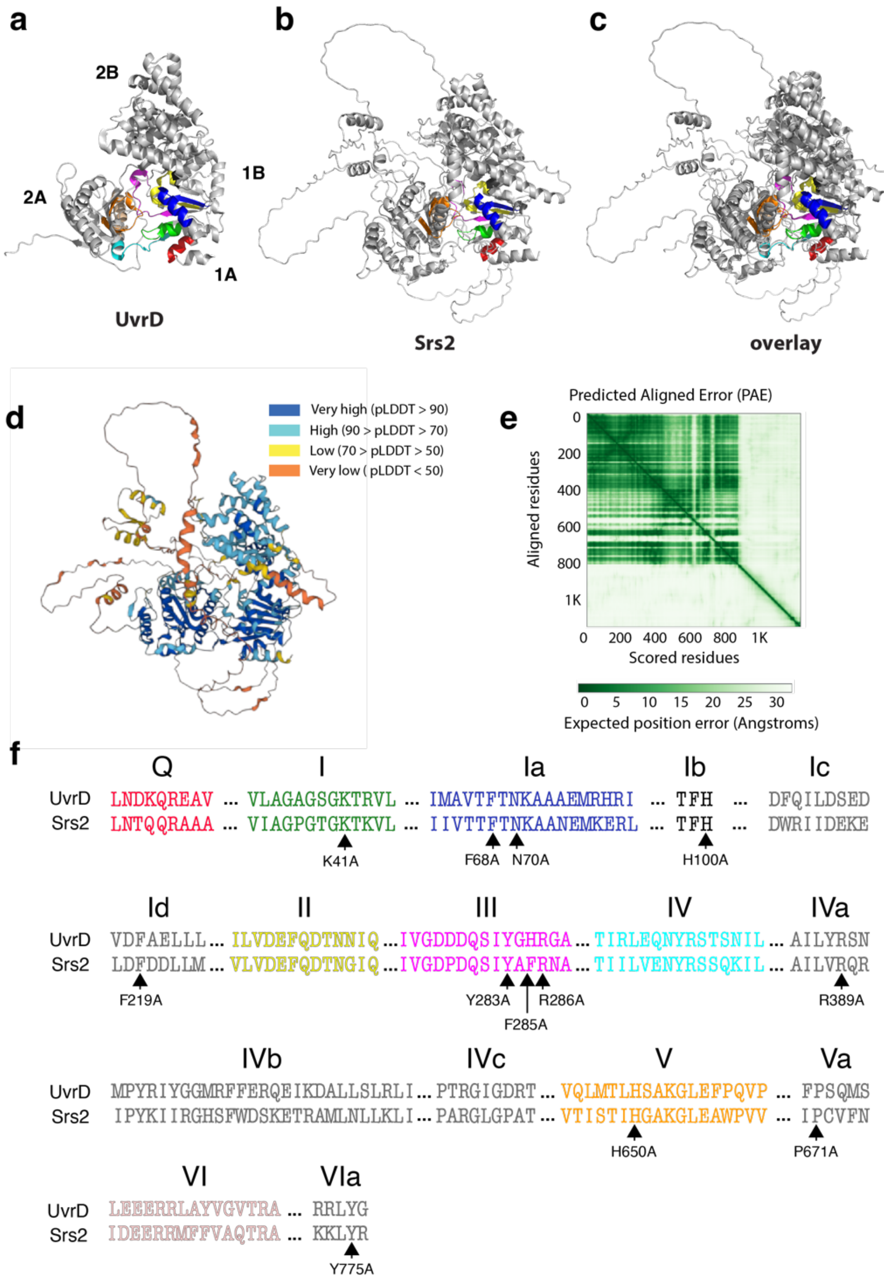

Supplementary Fig. 1. Alignment of Srs2 helicase motifs. a Crystal structure of UvrD (PDB ID 2IS1). **b** AlphaFold model for *S. cerevisiae* Srs2. **c** Merged UvrD crystal structure with the AlphaFold model of Srs2. **d** Per-residue model confidence score (pLDDT) plot for the Srs2 model as generated by Alphafold; regions below 50 pLDDT may be unstructured in isolation. **e** Predicted aligned error plot for the Srs2 model as generated by Alphafold. **f** Primary sequence alignment of Srs2 and UvrD. In **a, b, c** and **f** the helicase motifs are color coded and in **d** the amino acid residues chosen for mutagenesis are highlighted with arrowheads.

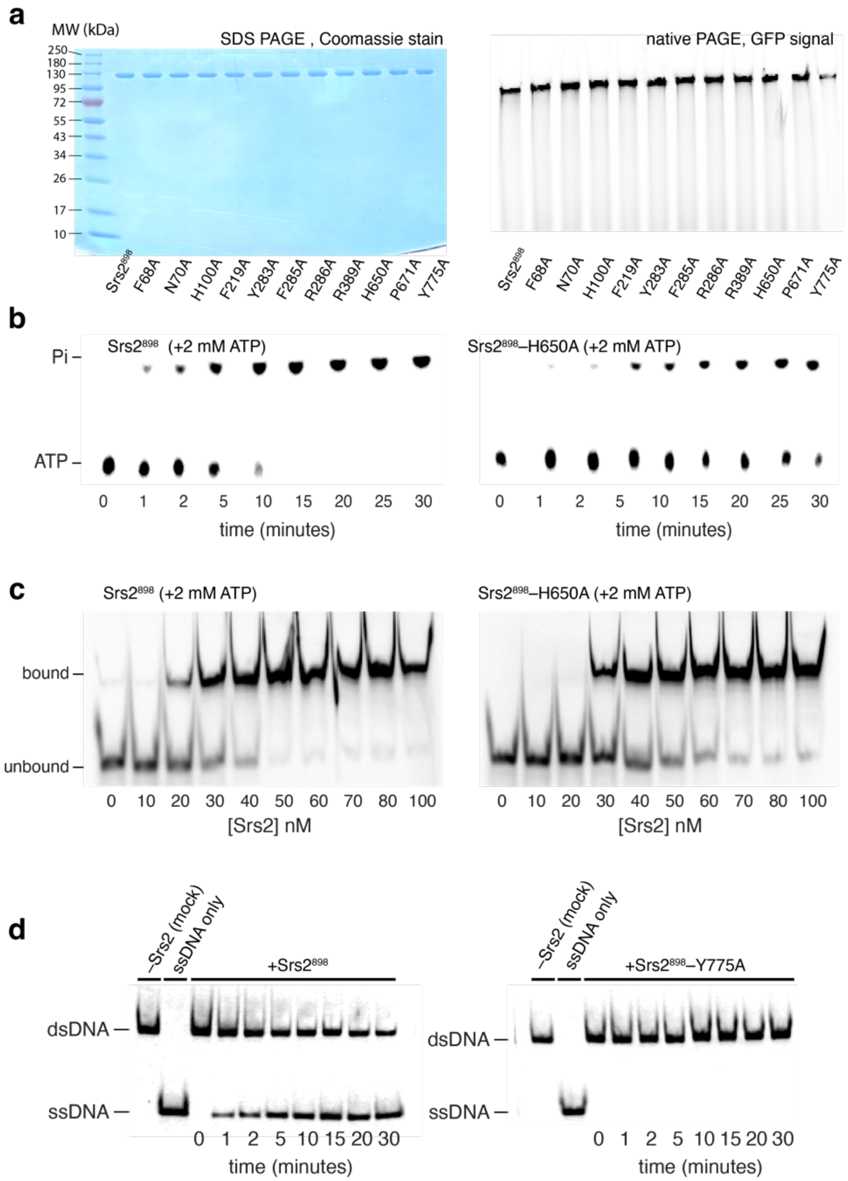

Supplementary Fig. 2. Examples of bulk biochemical assays. **a** SDS PAGE with Coomassie staining (left panel) and native PAGE scanned for GFP fluorescence (right panel) for all of the Srs2^{898} and mutant protein used in this study. **b** Images of TLC plates used for ATP hydrolysis assays for Srs2^{898} or Srs2^{898}–H650A, as indicated. **c** EMSA assays for ssDNA binding showing the unbound and bound ssDNA in reactions with Srs2^{898} or Srs2^{898}–H650A, as indicated. **d** Helicase assays for Srs2^{898} and Srs2^{898}–Y775A (40 nM protein) showing the full-length dsDNA (Alexa647 labeled 40–bp dsDNA with a 40–nt 3′ ssDNA overhang) and the unwound ssDNA product.

a

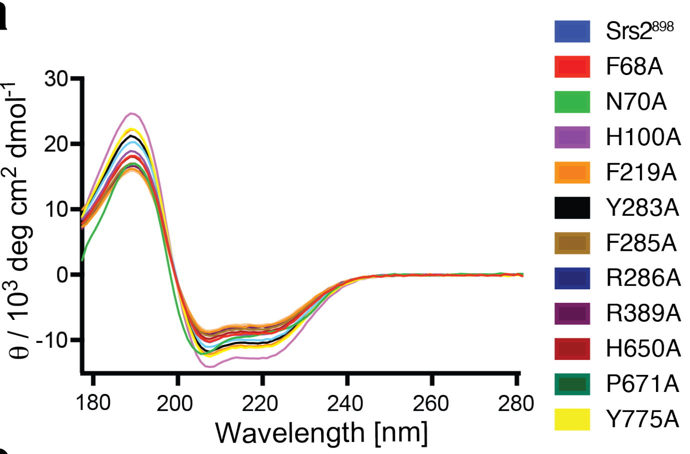

b

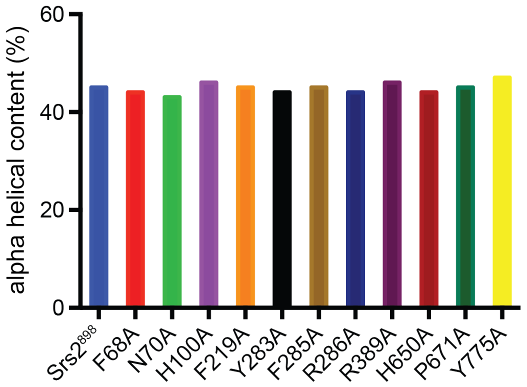

Supplementary Fig. 3. CD Spectra for all Srs2^{898} proteins used in this study. a Overlaid CD spectra for all of the GFP-tagged Srs2^{898} mutants used in this study. Each trace represents the average of ten scans. **b** Bar graph showing the alpha helical content for all the Srs2^{898} mutants. Note, GFP–Srs2^{898} is predicted to contain 41% helical content based upon the known structure of GFP and the AlphaFold model of Srs2, which is in good agreement with the experimental observations.

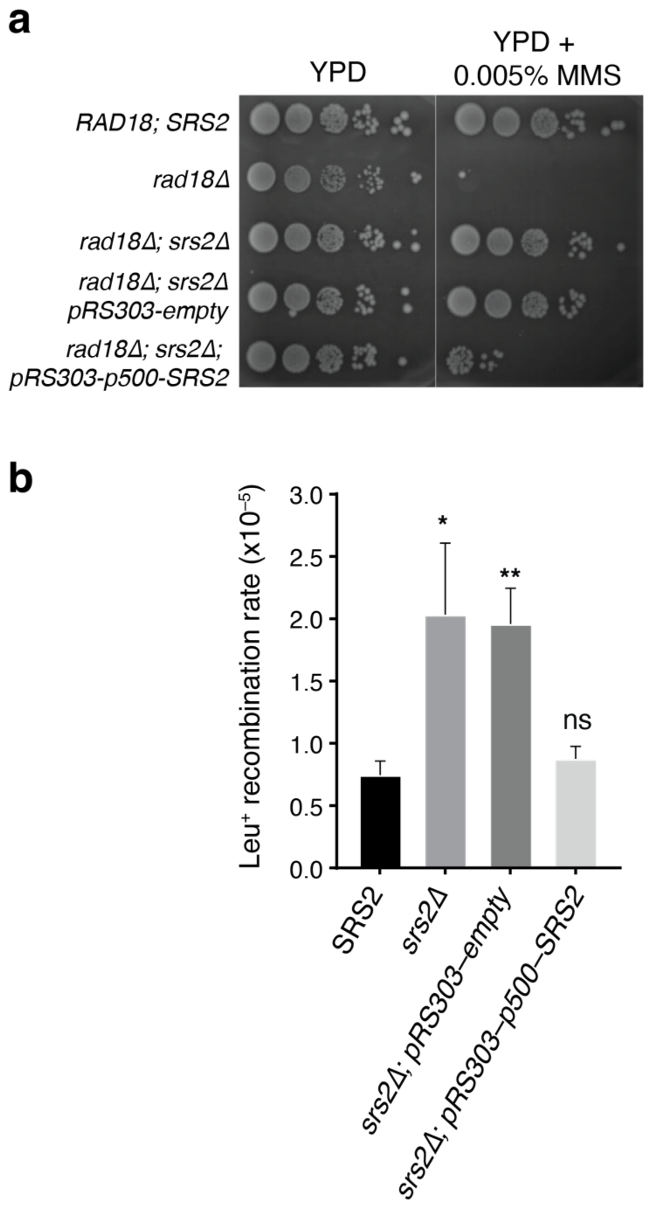

Supplementary Fig. 4. Control assays for MMS sensitivity and spontaneous recombination of the *SRS2* mutants. a Serially diluted spots of haploid yeast strains with indicated genotypes are shown on both YPD and YPD containing 0.005% MMS. Deletion of *SRS2* suppresses the *rad18Δ* associated MMS sensitivity. Incorporation of the integrative plasmid (pRS303) containing wild type *SRS2* along with its promoter renders the cells sensitive to MMS again (n=3). **b** Graph showing the spontaneous recombination rate determined from three independent fluctuation tests performed on the indicated strains. Asterisks represent statistical significance determined by unpaired t tests; ns (not significant).

a

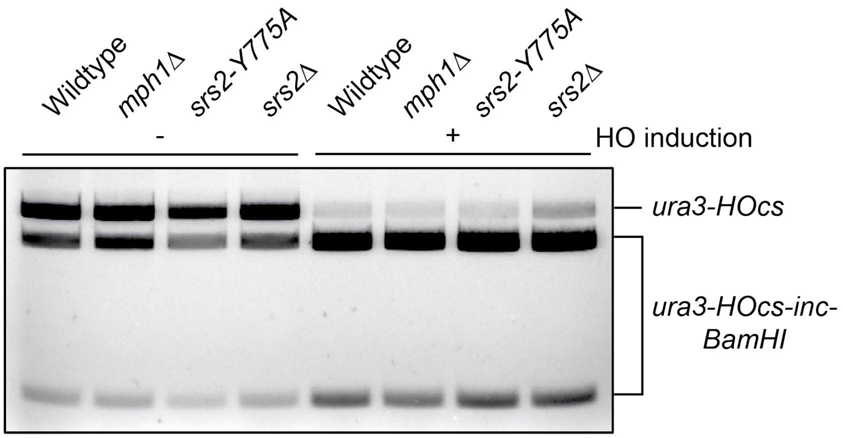

b

	%CO (after HO indiction)
Wildtype	10.66 ± 0.61
*mph1*Δ	22.01 ± 3.99 (p = 8.2×10^{-3})
srs2-Y775A	10.76 ± 2.31 (p = 0.944)
*srs2*Δ	19.64 ± 2.72 (p = 5.1×10^{-3})

c

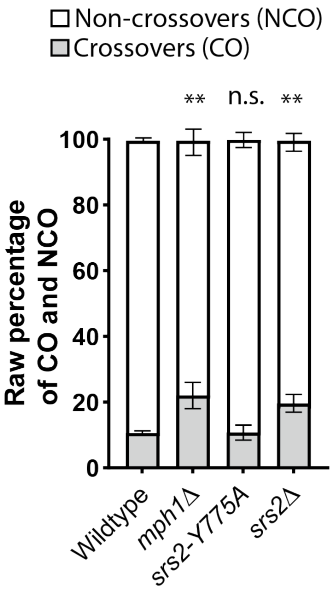

Supplementary Fig. 5. Control experiments depicting efficient repair by gene conversion. a The efficiency of repair in cells with the indicated genotype after HO cleavage by galactose induction determined by PCR amplification of the *ura3* loci followed by BamHI digestion. **b** Quantitated values for %CO formation after HO induction (n=3). **c** Percent distribution of recombinants, crossover (CO) and Non-crossover (NCO), shown for the indicated strains after HO induction.

Supplementary Table 1. Quantitation of Srs2[898] bulk biochemical data.

	**ATP hydrolysis (V_{max}; μM/sec)**	**ATP hydrolysis (K_M; mM)**	**ATP hydrolysis (k_{cat}; sec^{-1})**	**ssDNA binding (K_d; nM)**	**% Helicase activity (30 min)**
**WT**	2.02 ± 0.31	1.58 ± 0.71	51 ± 8	29 ± 3	100
**F68A**	1.95 ± 0.31 (P = 0.971)	1.52 ± 0.71 (P = 0.989)	49 ± 8 (P = 0.968)	31 ± 2 (P = 0.975)	92 ± 7 (P = 0.891)
**N70A**	1.87 ± 0.29 (P = 0.932)	1.32 ± 0.67 (P = 0.801)	47 ± 7 (P = 0.975	30 ± 3 (P = 0.921)	97 ± 2 (P =0.913)
**H100A**	1.81 ± 0.3 (P = 0.924)	1.33 ± 0.71 (P = 0.819)	45 ± 8 (P = 0.917)	31 ± 3 (P = 0.896)	79 ± 6 (P < 0.01)
**F219A**	1.66 ± 0.24 (P = 0.715)	1.51 ± 0.66 (P = 0.978)	42 ± 6 (P = 0.69)	31 ± 4 (P = 0.888)	55 ± 7 (P <0.0001)
**Y283A**	1.76 ± 0.25 (P = 0.812)	1.52 ± 0.63 (P = 0.982)	44 ± 6 (P = 0.791)	32 ± 3 (P = 0.903)	75 ± 8 (P < 0.001)
**F285A**	1.03 ± 0.16 (P <0.001)	2.75 ± 0.79 (P < 0.001)	25 ± 4 (P < 0.001)	41 ± 4 (P = 0.55)	25 ± 3 (P < 0.0001)
**R286A**	1.67 ± 0.26 (P = 0.587)	1.59 ± 0.71 (P = 0.964)	42 ± 6 (P = 0.674)	31 ± 2 (P = 0.878)	99 ± 1 (P = 0.991)
**R389A**	1.78 ± 0.28 (P = 0.754)	1.6 ± 0.72 (P = 0.943)	44 ± 7 (P = 0.776)	36 ± 3 (P = 0.772)	70 ± 4 (P < 0.001)
**H650A**	0.59 ± 0.09 (P <0.001)	2.91 ± 0.78 (P < 0.001)	15 ± 2 (P < 0.001)	46 ± 3 (P = 0.47)	28 ± 3 (P < 0.0001)
**P671A**	2.02 ± 0.31 (P = 0.999)	1.59 ± 0.72 (P = 0.966)	50 ± 7 (P = 0.995)	38 ± 2 (P = 0.75)	82 ± 4 (P < 0.01)
**Y775A**	2.01 ± 0.32 (P = 0.995)	1.61 ± 0.74 (P = 0.939)	51 ± 8 (P = 0.984)	30 ± 3 (P = 0.9)	3 ± 2 (P < 0.0001)

†Note that the results are derived from three independent experiments and p-values for the significance of differences in mean values were calculated by unpaired t-test (two sided) are indicated in each tab.

Supplementary Table 2. Comparison of relative activity levels for all Srs2^{898} mutants compared to wild type.

	K_M for ATP hydrolysis	V_{max} for ATP hydrolysis	k_{cat} for ATP hydrolysis	K_d for ssDNA binding	Helicase activity	Velocity on Rad51-ssDNA	Processivity on Rad51-ssDNA	Velocity on RPA-ssDNA	Processivity on RPA-ssDNA
**WT**	1.58 mM	2.02 μM/sec	51 sec^{-1}	29 nM	1	146 nt/sec	19 knt	179 nt/sec	15.4 knt
**F68A**	–3.8%	–3.5%	–3.9%	–6.9%	–14%	–16%	–17%	–34%	–17%
**N70A**	–16%	–7.4%	–7.8%	–3.4%	0%	–6.2%	+4.2%	–4.5%	–7.1%
**H100A**	–16%	10%	–12%	–6.9%	–25%	–39%	–13%	–52%	–44%
**F219A**	–4.4%	–18%	–18%	–6.9%	–48%	–48%	–54%	–56%	–64%
**Y283A**	–3.8%	–13%	–14%	–10%	–31%	–35%	–23%	–54%	–45%
**F285A**	+74%	–49%	–51%	–41%	–91%	N.A.	N.A.	N.A.	N.A.
**R286A**	+0.63%	–17%	–18%	–6.9%	0%	–4.1%	+1.0%	–23%	–16%
**R389A**	+1.3%	–12%	–14%	–24%	–37%	–50%	–53%	–61%	–55%
**H650A**	+84%	–71%	–71%	–59%	–92%	N.A.	N.A.	N.A.	N.A.
**P671A**	+0.63%	0%	–2.0%	–31%	–28%	–41%	–35%	–51%	–47%
**Y775A**	–1.9%	–0.5%	0%	–3.4%	–92%	–5.5%	+2.6%	–8.9%	–7.1%

All values for the Srs2 point mutants represent percent change relative to the values shown for GFP–Srs2^{898} (labeled as WT) in the first row.

The single molecule translocation and processivity data F285 and H650 are listed as N.A. (not applicable) because no translocation was observed for either mutant on either Rad51–ssDNA or RPA–ssDNA.

Supplementary Table 3A. GFP–Srs2^{898} translocation parameters on Rad51–ssDNA.

	Velocity (nt/sec)	Distance (knts)	N value
**WT†**	146 ± 50	19 ± 8.8	81
**F68A**	122 ± 43 (p = 0.0024)	15.8 ± 6.6 (p = 0.0131)	74
**N70A**	137 ± 46 (p = 0.3025)	19.8 ± 9.0 (p = 0.5614)	74
**H100A**	89 ± 40 (p < 0.0001)	16.5 ± 9.1 (p = 0.0848)	70
**F219A**	76 ± 27 (p < 0.0001)	8.8 ± 5.2 (p < 0.0001)	66
**Y283A**	95 ± 27 (p < 0.0001)	14.7 ± 9.7 (p = 0.0031)	78
**F285A**	0	0	76
**R286A**	140 ± 41 (p = 0.4760)	19.2 ± 8.8 (p = 0.8786)	66
**R389A**	73 ± 27 (p < 0.0001)	9 ± 4.5 (p < 0.0001)	75
**H650A**	0	0	68
**P671A**	86 ± 22 (p < 0.0001)	12.3 ± 7.1 (p < 0.0001)	59
**Y775A**	138 ± 37 (p = 0.3192)	19.5 ± 9.7 (p = 0.7420)	72

†Note that "WT" refers to GFP–Srs2^{898}; the results are derived from three independent experiments and p-values for the significance of differences in mean values were calculated by unpaired t-test (two sided) are indicated in each tab.

Supplementary Table 3B. GFP–Srs2^{898} translocation parameters on RPA–ssDNA.

	Velocity (nt/sec)	Distance (knts)	N value
**WT†**	179 ± 61	15.4 ± 4.3	76
**F68A**	119 ± 39 (p < 0.0001)	12.8 ± 4.2 (p = 0.0002)	78
**N70A**	171 ± 41 (p = 0.4114)	14.3 ± 4.3 (p = 0.1367)	72
**H100A**	86 ± 26 (p < 0.0001)	8.7 ± 2.8 (p < 0.0001)	72
**F219A**	79 ± 22 (p < 0.0001)	5.5 ± 1.9 (p < 0.0001)	82
**Y283A**	83 ± 23 (p < 0.0001)	8.5 ± 3.8 (< 0.0001)	78
**F285A**	0	0	70
**R286A**	138 ± 58 (p <0.0001)	13 ± 4.8 (p = 0.0024)	64
**R389A**	70 ± 33 (p < 0.0001)	6.9 ± 3.7 (p < 0.0001)	56
**H650A**	0	0	52
**P671A**	88 ± 32 (p < 0.0001)	8.2 ± 4.7 (p < 0.0001)	55

†Note that "WT" refers to GFP–Srs2^{898}; the results are derived from three independent experiments and p-values for the significance of differences in mean values were calculated by unpaired t-test (two sided) are indicated in each tab.

Supplementary Table 4. Yeast strains (All yeast strains are of W303 background).

Strain designation	Genotype
yECG04	*MATα ADE2 leu2-3,112 his3-11,15 ura3-1 LYS2 trp1-1 RAD5 MET15*
yECG06	*MATα rad18::HphMX leu2-3,112 his3-11,15 ura3-1 lys2Δ TRP1*
yECG07	*MATα ADE2 leu2-ΔEcoRI::URA3::leu2-DBstEII TRP1 lys2Δ RAD5*
yECG13	*MATα srs2::KanMX rad18::HphMX ADE2 leu2-3,112 his3-11,15 ura3-1 trp1-1 lys2Δ RAD5 MET15*
yECG14	*MATα ADE2 srs2::KanMX his3-11,15 leu2-ΔEcoRI::URA3::leu2-DBstEII trp1-1 lys2Δ RAD5*
yECG16	*MATα srs2::KanMX, rad18::HphMX, his3-11,15::pRS303-empty::HIS3, ADE2, leu2-3,112, ura3-1, trp1-1, lys2Δ, RAD5 MET15*
yECG17	*MATα ADE2 srs2::KanMX his3-11,15::pRS303vanilla::HIS3, leu2-ΔEcoRI::URA3::leu2-DBstEII trp1-1 lys2Δ RAD5*
yECG18	*MATα srs2::KanMX, rad18::HphMX, his3-11,15::pRS303-p500-SRS2WT::HIS3, ADE2, leu2-3,112, ura3-1, trp1-1, lys2Δ, RAD5 MET15*
yECG19	*MATα ADE2 srs2::KanMX his3-11,15::pRS303-p500-SRS2WT::HIS3, leu2-ΔEcoRI::URA3::leu2-DBstEII trp1-1 lys2Δ RAD5*
yECG20	*MATα srs2::KanMX rad18::HphMX ADE2 leu2-3,112 his3-11,15::pRS303-p500-SRS2-F68A::HIS3 ura3-1 trp1-1 lys2Δ RAD5 MET15*
yECG21	*MATα srs2::KanMX rad18::HphMX ADE2 leu2-3,112 his3-11,15::pRS303-p500-SRS2-N70A::HIS3 ura3-1 trp1-1 lys2Δ RAD5 MET16*
yECG22	*MATα srs2::KanMX rad18::HphMX ADE2 leu2-3,112 his3-11,15::pRS303-p500-SRS2-H100A::HIS3 ura3-1 trp1-1 lys2Δ RAD5 MET17*
yECG23	*MATα srs2::KanMX rad18::HphMX ADE2 leu2-3,112 his3-11,15::pRS303-p500-SRS2-F219A::HIS3 ura3-1 trp1-1 lys2Δ RAD5 MET18*
yECG24	*MATα srs2::KanMX rad18::HphMX ADE2 leu2-3,112 his3-11,15::pRS303-p500-SRS2-Y283A::HIS3 ura3-1 trp1-1 lys2Δ RAD5 MET19*
yECG25	*MATα srs2::KanMX rad18::HphMX ADE2 leu2-3,112 his3-11,15::pRS303-p500-SRS2-F285A::HIS3 ura3-1 trp1-1 lys2Δ RAD5 MET20*
yECG26	*MATα srs2::KanMX rad18::HphMX ADE2 leu2-3,112 his3-11,15::pRS303-p500-SRS2-R286A::HIS3 ura3-1 trp1-1 lys2Δ RAD5 MET21*
yECG27	*MATα srs2::KanMX rad18::HphMX ADE2 leu2-3,112 his3-11,15::pRS303-p500-SRS2-R389A::HIS3 ura3-1 trp1-1 lys2Δ RAD5 MET22*

yECG28	*MATα srs2::KanMX rad18::HphMX ADE2 leu2-3,112 his3-11,15::pRS303-p500-SRS2-H650A::HIS3 ura3-1 trp1-1 lys2Δ RAD5 MET23*
yECG29	*MATα srs2::KanMX rad18::HphMX ADE2 leu2-3,112 his3-11,15::pRS303-p500-SRS2-P671A::HIS3 ura3-1 trp1-1 lys2Δ RAD5 MET24*
yECG30	*MATα srs2::KanMX rad18::HphMX ADE2 leu2-3,112 his3-11,15::pRS303-p500-SRS2-Y775A::HIS3 ura3-1 trp1-1 lys2Δ RAD5 MET25*
yECG35	*MATα ADE2 srs2::KanMX his3-11,15::pRS303-p500-SRS2-F68A::HIS3, leu2-ΔEcoRI::URA3::leu2-DBstEII trp1-1 lys2Δ RAD5*
yECG36	*MATα ADE2 srs2::KanMX his3-11,15::pRS303-p500-SRS2-N70A::HIS3, leu2-ΔEcoRI::URA3::leu2-DBstEII trp1-1 lys2Δ RAD5*
yECG37	*MATα ADE2 srs2::KanMX his3-11,15::pRS303-p500-SRS2-H100A::HIS3, leu2-ΔEcoRI::URA3::leu2-DBstEII trp1-1 lys2Δ RAD5*
yECG38	*MATα ADE2 srs2::KanMX his3-11,15::pRS303-p500-SRS2-F219A::HIS3, leu2-ΔEcoRI::URA3::leu2-DBstEII trp1-1 lys2Δ RAD5*
yECG39	*MATα ADE2 srs2::KanMX his3-11,15::pRS303-p500-SRS2-Y283A::HIS3, leu2-ΔEcoRI::URA3::leu2-DBstEII trp1-1 lys2Δ RAD5*
yECG40	*MATα ADE2 srs2::KanMX his3-11,15::pRS303-p500-SRS2-F285A::HIS3, leu2-ΔEcoRI::URA3::leu2-DBstEII trp1-1 lys2Δ RAD5*
yECG41	*MATα ADE2 srs2::KanMX his3-11,15::pRS303-p500-SRS2-R286A::HIS3, leu2-ΔEcoRI::URA3::leu2-DBstEII trp1-1 lys2Δ RAD5*
yECG42	*MATα ADE2 srs2::KanMX his3-11,15::pRS303-p500-SRS2-R389A::HIS3, leu2-ΔEcoRI::URA3::leu2-DBstEII trp1-1 lys2Δ RAD5*
yECG43	*MATα ADE2 srs2::KanMX his3-11,15::pRS303-p500-SRS2-H650A::HIS3, leu2-ΔEcoRI::URA3::leu2-DBstEII trp1-1 lys2Δ RAD5*
yECG44	*MATα ADE2 srs2::KanMX his3-11,15::pRS303-p500-SRS2-P671A::HIS3, leu2-ΔEcoRI::URA3::leu2-DBstEII trp1-1 lys2Δ RAD5*
yECG45	*MATα ADE2 srs2::KanMX his3-11,15::pRS303-p500-SRS2-Y775A::HIS3, leu2-ΔEcoRI::URA3::leu2-DBstEII trp1-1 lys2Δ RAD5*
yECG63	*MATa-inc ura3::HOcs lys2::ura3-HOcs inc (5.6 kb) ade3::GAL-HO srs2::KanMX his3::pRS303-p500-SRS2-775A::HIS3 leu2-3, 112 trp1-1 ade2-1(?) can1-100 RAD5*
yECG64	*MATa-inc ura3::HOcs lys2::ura3-HOcs inc (5.6 kb) ade3::GAL-HO srs2::KanMX LEU2 trp1-1 ade2-1(?) can1-100 RAD5*

yECG70	*MATα srs2Δ:NatMX, his3::pRS303-p500-SRS2-775A::HIS3, ADE2, leu2-3,112, ura3-1, TRP1, lys2Δ, RAD5 MET15*
LSY2520	*MATa-inc ura3::HOcs lyss2::ura3-HOcs inc (5.6 kb) ade3::GAL-HO his3-11, 15 leu2-3, 112 trp1-1 ade2-1 can1-100 RAD5*
LSY2598-34B	*MATa-inc ura3::HOcs lys2::ura3-HOcs inc (5.6 kb) ade3::GAL-HO mph1::KanMX6 his3-11, 15 leu2-3, 112 trp1-1 ade2-1 can1-100 RAD5*
LSY0403	*MATa rad54Δ::LEU2, RAD5, trp1-1, his3-11,15, ura3-1, ade2-1*
LSY1975	*MATa sgs1Δ::HPHMX4, RAD5, leu2-3,112, trp1-1, ura3-1, his3-11,15, ade2-1*
LSY2330	*MATa mph1Δ::KANMX, RAD5, ADE2, met17-s, trp1-1, his3-11,15, ura3-1, leu2-3,112*
