# Supplementary material for: The separation pin distinguishes the pro– and anti–recombinogenic functions of Saccharomyces cerevisiae Srs2
Source: Nat Commun. 2023 Dec 8;14:8144. doi: 10.1038/s41467-023-43918-4 (PMC10709652; doi:10.1038/s41467-023-43918-4)
Supplement: Supplementary file 3 — Reporting Summary [file 41467_2023_43918_MOESM3_ESM.pdf]

Corresponding author(s): Eric Greene
Last updated by author(s): 2023 - 11 - 05

Reporting Summary

Nature Portfolio wishes to improve the reproducibility of the work that we publish. This form provides structure for consistency and transparency in reporting. For further information on Nature Portfolio policies, see our Editorial Policies and the Editorial Policy Checklist.

Statistics

For all statistical analyses, confirm that the following items are present in the figure legend, table legend, main text, or Methods section.

n/a	Confirmed	
☐	☒	The exact sample size (*n*) for each experimental group/condition, given as a discrete number and unit of measurement
☐	☒	A statement on whether measurements were taken from distinct samples or whether the same sample was measured repeatedly
☐	☒	The statistical test(s) used AND whether they are one- or two-sided<br>*Only common tests should be described solely by name; describe more complex techniques in the Methods section.*
☐	☒	A description of all covariates tested
☐	☒	A description of any assumptions or corrections, such as tests of normality and adjustment for multiple comparisons
☐	☒	A full description of the statistical parameters including central tendency (e.g. means) or other basic estimates (e.g. regression coefficient) AND variation (e.g. standard deviation) or associated estimates of uncertainty (e.g. confidence intervals)
☐	☒	For null hypothesis testing, the test statistic (e.g. *F*, *t*, *r*) with confidence intervals, effect sizes, degrees of freedom and *P* value noted<br>*Give P values as exact values whenever suitable.*
☒	☐	For Bayesian analysis, information on the choice of priors and Markov chain Monte Carlo settings
☒	☐	For hierarchical and complex designs, identification of the appropriate level for tests and full reporting of outcomes
☒	☐	Estimates of effect sizes (e.g. Cohen's *d*, Pearson's *r*), indicating how they were calculated

Our web collection on statistics for biologists contains articles on many of the points above.

Software and code

Policy information about availability of computer code

Data collection	Single molecule data were collected using NIS-Elements Version 5.11 (Nikon) microscope equipped with a 488-nm laser (Coherent Sappphire. 200mW), a 561-nm laser (Coherent Sapphire, 200mW), and two Andor iXon EMCCD cameras. Phosphor imaging and gels for bulk experiments were scanned using typhoon FLA 9000 phosphor imager (GE healthcare). For Southern blot, the signal was detected using Typhoon trio phosphoimager (GE healthcare).
Data analysis	Analysis of single molecule data and Southern blot data were performed using open-source image processing software ImageJ 1.53C (FIJI). All the graphs were plotted using GraphPad Prism version 6.

For manuscripts utilizing custom algorithms or software that are central to the research but not yet described in published literature, software must be made available to editors and reviewers. We strongly encourage code deposition in a community repository (e.g. GitHub). See the Nature Portfolio guidelines for submitting code & software for further information.

Data

Policy information about availability of data

All manuscripts must include a data availability statement. This statement should provide the following information, where applicable:

- Accession codes, unique identifiers, or web links for publicly available datasets
- A description of any restrictions on data availability
- For clinical datasets or third party data, please ensure that the statement adheres to our policy

All the information generated and analyzed is included in the manuscript and all graphs have associated raw data that is provided as an Excel worksheet organized by figures (Source Data file 1). Full scans of all gels are also provided as Source data (Source Data file 1). Kymographs used for all single molecule data analysis are also provided (Source Data file 1). Source data are provided with this paper.

Research involving human participants, their data, or biological material

Policy information about studies with human participants or human data. See also policy information about sex, gender (identity/presentation), and sexual orientation and race, ethnicity and racism.

Reporting on sex and gender	Not applicable. No human participants, their data or biological material were used/involved in this study.
Reporting on race, ethnicity, or other socially relevant groupings	Not applicable. No human research participants were involved.
Population characteristics	Not applicable. No human research participants were involved.
Recruitment	not applicable. no participants were recruited for this study.
Ethics oversight	Not applicable. there was no need for approval from the relevant organization for this study.

Note that full information on the approval of the study protocol must also be provided in the manuscript.

Field-specific reporting

Please select the one below that is the best fit for your research. If you are not sure, read the appropriate sections before making your selection.

☒ Life sciences ☐ Behavioural & social sciences ☐ Ecological, evolutionary & environmental sciences

For a reference copy of the document with all sections, see nature.com/documents/nr-reporting-summary-flat.pdf

Life sciences study design

All studies must disclose on these points even when the disclosure is negative.

Sample size	Sample sizes were not pre-selected. All single molecule measurements reflect the cumulative results from tens to hundreds of individual molecules, which is consistent with expectations of the field (all exact n values and associated statistical parameters are reported in the manuscript). For direct repeat recombination assay, recombination rate for each genotype was determined three times by performing fluctuation test on eight independent colonies (consistent with the expectation of the field).
Data exclusions	Data were not excluded
Replication	All reported data represent a minimum of three replicates to ensure reproducibility
Randomization	Randomization was not part of the study design
Blinding	Blinding was not part of the study design. This research involved molecular analysis of specific protein mutants and the researchers needed to make and characterize each known mutant independently

Reporting for specific materials, systems and methods

We require information from authors about some types of materials, experimental systems and methods used in many studies. Here, indicate whether each material, system or method listed is relevant to your study. If you are not sure if a list item applies to your research, read the appropriate section before selecting a response.

Materials & experimental systems

n/a	Involved in the study
☒	☐ Antibodies
☒	☐ Eukaryotic cell lines
☒	☐ Palaeontology and archaeology
☒	☐ Animals and other organisms
☒	☐ Clinical data
☒	☐ Dual use research of concern
☒	☐ Plants

Methods

n/a	Involved in the study
☒	☐ ChIP-seq
☒	☐ Flow cytometry
☒	☐ MRI-based neuroimaging
